# Protocol for quantifying *Drosophila* feeding behavior using flyPAD and optoPAD

**DOI:** 10.1016/j.xpro.2026.104711

**Published:** 2026-07-21

**Authors:** Nicholas J. Collins, Madison N. Endres, Irina T. Sinakevitch, Lisha Shao

**Affiliations:** 1 Department of Biological Sciences, University of Delaware, Newark, DE, USA

**Keywords:** Behavior, High Throughput Screening, Model Organisms

## Abstract

Quantifying feeding behavior with precision is essential for understanding how internal state, sensory cues, and neural activity influence food intake and dietary choice. Here, we present a protocol for performing consumption and dietary choice assays in *Drosophila* using the flyPAD/optoPAD system. We provide instructions for food preparation, arena setup, data acquisition, and analysis for studying feeding behavior, nutrient preference, and learning. This approach enables the measurement of feeding events across multiple arenas while allowing precise control of food substrates and optogenetic stimulation.

For complete details on the use and execution of this protocol, please refer to Christie et al.^1^

## Highlights

• Assemble and validate flyPAD/optoPAD arenas for capacitance-based feeding assays
• Configure flyPAD assays and open- or closed-loop optoPAD stimulation paradigms
• Quantify total consumption, two-choice preference, and operant-style learned preference
• Compute sip-, bout-, and burst-level metrics with appropriate statistical tests

## Abstract (editor highlights)

Publisher’s note: Undertaking any experimental protocol requires adherence to local institutional guidelines for laboratory safety and ethics.

## Graphical abstract

**Figure:**
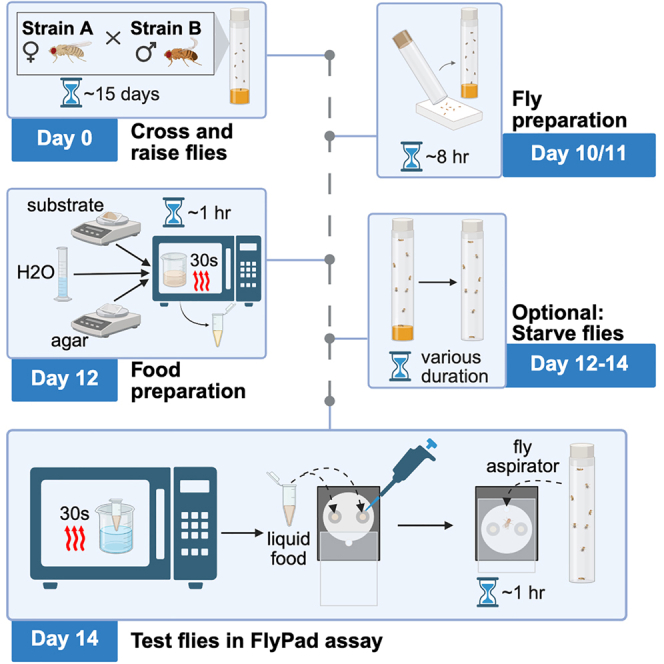

## Before you begin

### Background

Feeding behavior in *Drosophila* is a widely used and biologically informative variable for understanding how genetic factors, cellular mechanisms, neural circuits, and internal physiological states interact to shape behavior. Alterations in feeding have been used to characterize the effects of social stress,^2^ investigate how nutrient valuation contributes to metabolic disease and obesity,^3^^,^^4^ map associative learning circuits that guide food choice,^1^^,^^5^ and reveal links between dietary intake, neurodegeneration, and oxidative stress.^6^ Because feeding behavior and dietary choice integrate sensory evaluation, motivation, reward, and metabolic demand, disruptions in this behavior often signal underlying changes in neuronal function.

Accordingly, identifying the neural circuits and cellular processes that regulate feeding has become a major research focus. Manipulating these pathways can rescue aberrant feeding phenotypes,^7^^,^^8^^,^^9^^,^^10^ and offers a tractable window into how physiological state modulates decision-making in the fly brain.^11^^,^^12^ However, many existing feeding assays lack the temporal precision or neural specificity needed to dissect how circuit activity dynamically shapes feeding decisions. The flyPAD/optoPAD assay addresses these gaps.

As outlined below, flyPAD/optoPAD provides high-resolution detection of feeding on solid food while enabling closed-loop, real-time optogenetic manipulation, making it well suited to reveal how specific neurons influence moment-to-moment feeding behavior.

### Important definitions

**flyPAD:** The experimental unit consists of a flyPAD feeding multiplexor device connected to flyPAD arenas, enabling high-resolution, capacitance-based detection of feeding on solid food substrates. The system can be used to measure total consumption and/or feeding preference between two substrates.^13^

**optoPAD:** Provides the same utility as the flyPAD, with the addition of an optogenetic module for open-loop and closed-loop assays.

**Open-loop**: A type of optoPAD experiment where optogenetic stimulation is delivered on a continuous, fixed schedule independent of the animal’s behavior.

**Closed-loop**: A type of optoPAD experiment where optogenetic stimulation is delivered contingent on the animal’s behavior. The fly’s own behavior facilitates feedback from the optogenetic module.^14^

Node: A node refers to an individual functional unit within the Bonsai software that performs a specific task, such as acquiring data, processing signals, detecting events, controlling hardware, or assigning output directory files.

### Innovation

The flyPAD/optoPAD feeding assay provides several advantages over existing feeding paradigms, including Con-Ex (blue dye), CAFE, and FLIC.^13^^,^^15^^,^^16^^,^^17^ The flyPAD/optoPAD system combines high-resolution, capacitance-based detection of feeding on solid food with closed-loop, millisecond-precision optogenetic control, enabling neural circuit perturbations that occur strictly during active feeding bouts. Compared to Con-Ex, which provides accurate but low-resolution measurements of total consumption, or FLIC, which offers high-resolution detection of interactions with liquid-food, the flyPAD/optoPAD system allows researchers to causally link neuronal activity to microstructural feeding decisions in real time on solid food.

This integration of precise behavioral readout and temporally locked circuit manipulation makes the flyPAD/optoPAD system well suited for dissecting how specific neurons drive feeding preference, consummatory microstructure, and the moment-to-moment dynamics of ingestive behavior.

### Institutional permissions

There are no specific institutional permissions required, other than standard bio-safety training as directed by local and federal requirements.

### Step-by-step preparation

The sections below highlight step-by-step preparation instructions for the procurement and setup of the flyPAD and optoPAD units (steps 1-4), cooking food and preparing flies for husbandry (steps 5-7), preparing retinal food for optogenetic experiments (steps 8-9), preparing experimental flies (steps 11-13) and food used for choice experiments (steps 14-15).

### Procurement and setup of flyPAD units and optoPAD LED modules

**Timing: 1–2 months prior to start of experiment**

This section describes step-by-step acquisition, initial setup, and pre-experimental validation of the flyPAD/optoPAD system. Early setup is critical to allow sufficient time for hardware troubleshooting, software configuration, and pilot testing prior to data collection. For detailed specifications, a user guide is provided from the manufacturer (FlyPad user guide, v01) upon procurement of the flyPAD units. For detailed information about the flyPAD units, see Table 1.

1. Procurement
  a. Obtain flyPAD units, sensor boards, control electronics, optoPAD optogenetic LED modules, power supplies, and all required cables from the manufacturer.
  b. Confirm with manufacturer that purchased units are compatible with both open-loop and closed-loop optogenetic configurations if desired.
  c. Ensure that replacement units, covers, screws, and cables are available prior to beginning experiments.
2. Initial hardware setup: flyPAD
  a. Place the flyPAD feeding multiplexor device on a vibration-minimized, level benchtop in a temperature- and humidity-controlled behavior testing room.
  b. Connect the flyPAD feeding multiplexor device to the desired laboratory computer using a micro-USB cable.
  c. Connect flyPAD arenas to the feeding multiplexor device using ribbon cables provided by the manufacturer.
    i. Secure flyPAD arenas evenly to the multiplexor, ensuring that all contact points are flush and screws are tightened uniformly to prevent signal drift from loose connections.
    ii. Confirm that the flyPAD arenas are level using a level.
3. Initial hardware setup: optoPAD
  a. Connect the optoPAD LED controller device to the desired laboratory computer using a micro-USB cable.
  b. Connect the LED add-on module to the LED controller using the manufacturer provided ribbon cables.
4. Software installation and configuration
  a. Install Bonsai (behavioral acquisition software) and required flyPAD/optoPAD packages on the acquisition computer as per manufacturer provided instructions.
    ***Note:*** The correct version of Bonsai to install is provided by the manufacturer.
  b. Verify correct COM port assignments for flyPAD sensors and optoPAD controllers within Bonsai. Port numbers may vary between systems.
    i. On Windows systems, the devices will appear under “Ports (COM & LTP)” as a USB serial device corresponding to the connected acquisition hardware.
      ***Note:*** The correct port can be identified by disconnecting and reconnecting the hardware and observing the corresponding COM port that appears/disappears.
    ii. Within Bonsai, click on the “device” node, and choose the correct port name where the flyPAD device is connected to your computer.
      ***Note:*** COM ports assignments may change when the unit is connected to a different computer. When the correct COM is selected and the flyPAD is powered, “device” will change its name to “FlyPad”.
  c. Load open-loop and closed-loop Bonsai workflows and confirm that all nodes execute without errors.
    ***Note:*** After installing the manufacture provided workflows for flyPAD and/or optoPAD (see step 4a above), available workflows will appear upon opening Bonsai.
  d. Open Bonsai and select “file”, “Open Workflow”. Select the workflow for your desired application (open-loop or closed-loop).
    i. Select “OpenLoopSimple_Red.bonsai” for open-loop experiments.
    ii. Select “Optopad_B2_4_NewVersion_Blinking_V2.bonsai” for closed-loop experiments.
  e. Pre-experimental calibration and validation
    i. Run a baseline channel test with empty arenas to confirm stable capacitance signals across all channels (Figure 2).

**Figure 1:**
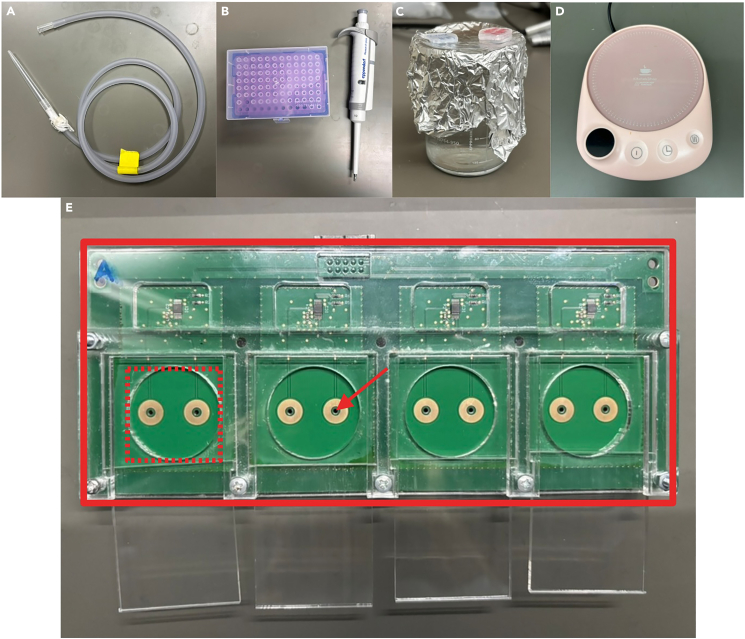
Required materials for the flyPAD assay (A) A fly aspirator is used to transfer a single fly into a flyPAD chamber. The aspirator is constructed from plastic tubing, cheese cloth, and a pipette tip. (B) A 10 μL pipette and tips are used to load the appropriate food media into each flyPAD channel (red arrow). Approximately 3 μL of media is loaded per channel. (C) Food media are stored in 5 mL Eppendorf tubes prior to heating. They are then heated and maintained in a hot water bath during the loading process. (D) A standard mug warmer is used to keep the food media in a liquid state between experiments, reducing the need for repeated reheating. (E) The flyPAD unit (solid red rectangle) is used for experimentation. One fly is loaded into each flyPAD chamber (dashed red rectangle). The transparent top panel of each chamber can slide open to allow loading of food media and flies during assay preparation. Created in BioRender. Endres, M. (2026) https://BioRender.com/kvzcpye.

**Figure 2:**
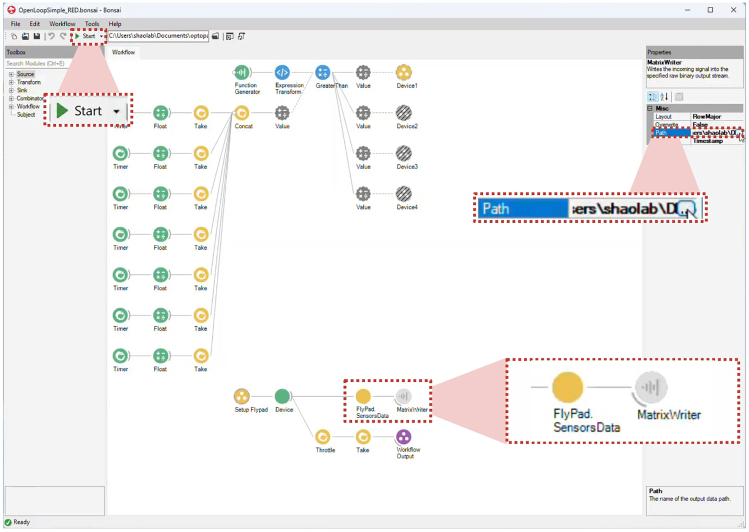
Key features of the Bonsai setup and assay initiation Shown is the typical appearance of a Bonsai workflow file. Critical parameters that should be edited prior to assay initiation are indicated by dashed boxes. “MatrixWriter” specifies where the Bonsai output file will be saved once the assay is stopped, and the “Path” field can be modified to direct the file to the appropriate storage location. “flyPADSensorsData” should be opened during media loading to confirm that all channels are functioning properly. After these settings have been configured for the experiment, the “Start” button can be clicked to initiate the assay. Created in BioRender. Endres, M. (2026) https://BioRender.com/rio6bu7.
  f. Load agar-only food (1% agar in distilled water) into test wells and initiate a short test recording to verify that all channels show dynamic, non-flat traces (see Figure 2; Figure 3).
    i. Click on “Start”
    ii. Double click on “flyPAD sensors data”.
    iii. Verify all lines are moving in a zig-zag pattern.

**Figure 3:**
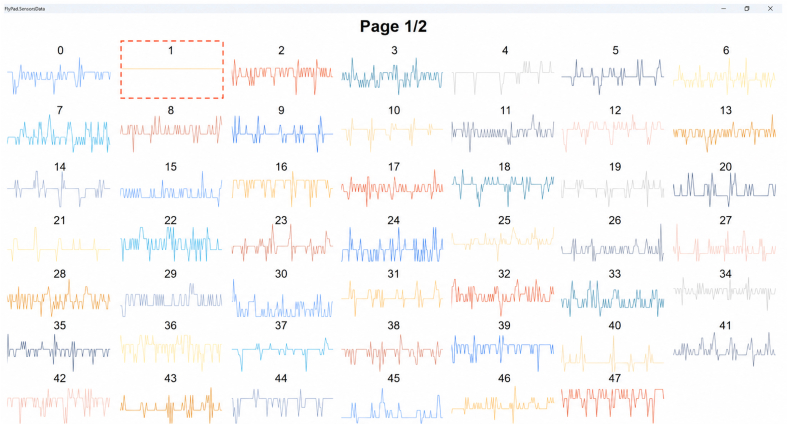
Example of flyPAD sensor data During loading of food media into the flyPAD channels, sensor data should be monitored to verify proper loading. Each panel corresponds to an individual flyPAD channel within a chamber and is numbered accordingly. After successful media loading, sensors should display a characteristic zig-zag pattern, indicating that sensor function has not been compromised by the media. In contrast, a flat signal (dashed box) suggests that media has spilled onto the sensor, which can interfere with subsequent data analysis. To avoid this issue, channels should be cleaned thoroughly and media loaded carefully. Once all sensors exhibit the zig-zag pattern, media loading is complete and the assay can be initiated.
  g. Temperature and Humidity
    i. Maintain testing room temperature at 22–25°C and relative humidity between 40-55%, as environmental variability can affect both feeding behavior and sensor sensitivity.
    ii. Conduct experiments in darkness or under controlled lighting conditions to prevent unintended visual stimulation.
      **CRITICAL:** Allocate sufficient time for pilot experiments to confirm reliable feeding engagement, sensor stability, and accurate synchronization of optogenetic stimulation before collecting experimental data.

**Table 1:** Equipment

Equipment	Source/Vendor	Notes/Purpose
FlyPAD 4-fly arena module	www.flypad.rocks	Each module has 8 sensitive electrodes, two per fly chamber. These allow precise detection of feeding behavior.
LED optogenetic add-on module	www.flypad.rocks	LED module for open and closed-loop optogenetic stimulation.
LED controller optogenetic add-on module	www.flypad.rocks	LED controller that gates power to the LEDs contingent on the real time feeding data (closed-loop) or preprogrammed stimulation pattern (open-loop stimulation).
FlyPAD feeding multiplexor	www.flypad.rocks	PC interface and an acquisition board for up to 12 of 4-fly arena modules.
Computer with Bonsai and flyPAD analysis software from the supplier	www.bonsai-rx.org	Bonsai for data acquisition, www.flypad.rocks provided software for data analysis.

### Cooking, dispensing, and storing standard food for husbandry

**Timing: 3–4 weeks prior to experiment; 1–2 h**

5. Cooking fly food for husbandry (base volume: 1 L). Scale as necessary.
  a. Add 1 L of distilled water and 200g of Nutri-FlyⓇ to a large pot.
  b. Place pot on an electric stove top, set on high heat (simmer setting, approximately between 85-96°C). Stir continuously to avoid food sticking to the bottom of the pot.
  c. Once the mixture reaches boiling point, put the burner to a higher heat (approximately 100°C), and continuously stir for an additional 10–15 min.
  d. Take off the food from the heat. Turn off the heat and allow the food to cool to 70°C with a continuous stir.
  e. Add propionic acid (4.8 mL) and Tegosept (10 mL) as preservatives. Stir food to evenly distribute ingredients.
6. Dispensing
  a. It is recommended to use a rapid dispensing system (see equipment below)
    i. For husbandry vials, fill to ∼2-3cm depth.
    ii. For collection vials (for collecting flies prior to beginning assay) fill to ∼1-2cm.
7. Storage
  a. Cover newly created food vials with cheese cloth and allow food to solidify overnight (6-8 h).
  b. Food can then be stored in 4°C for approximately 1 month or kept at room temperature (20–25°C) for up to 3–5 days.

***Note:*** Do not insert vial plugs during cooling to avoid condensation. Plugs should be inserted after the cooling period to avoid excessive drying of the food. Cover tightly with plastic film and cover in a plastic bag. Ensure there is no moisture on the walls of the vials when transferring flies to avoid fly drowning.

### Preparing retinal food for husbandry and optoPAD

**Timing: ∼2 weeks prior to experiment**

8. Cooking retinal food (optogenetic experiments)
  a. Prepare retinal stock solution
    i. Dissolve all-trans retinal in ethanol to make a 100 mM stock solution.
    ii. Prepare retinal stock solution under minimal light conditions and store the aliquots in dark conditions (amber vials or foil wrapped tubes).
  b. Follow the standard food preparation steps outlined above.
9. Addition of retinal to standard food
  a. Once food reaches a temperature of 60°C, turn off all the lights in the laboratory. Dim ambient lighting may be used.
  b. Add the appropriate amount of retinal.
    i. Husbandry vials: 1 mL retinal stock solution per 500 mL food.
    ii. Collection vials: 1 mL retinal stock solution per 250 mL food.
10. Repeat standard food protocol for cooling, dispensing, and storage.

**CRITICAL: 1:** Place retinal food into a light-sensitive plastic bag to prevent degradation.

**CRITICAL: 2:** You may prepare 0.2 mM retinal-containing food for husbandry (1:500 ratio). This is recommended if flies are collected from these vials and immediately used in behavioral experiments. For collection vials, you may use 0.4 mM retinal containing food (1:250 ratio), and flies should remain on this food for at least 3 days prior to the experiment.

### Preparing experimental flies

**Timing: ∼2 weeks prior to experiment**

11. Maintain newly acquired stocks (e.g., from the Bloomington Stock Center) in husbandry vials as described above in a 25°C incubator.
  a. Temperatures between 18°C to 27°C can be used to slow or speed up development, respectively.
  b. Fly incubators should be set to allow for a 12-hour dark/light cycle.
  c. Humidity should be set to 50-55%.
12. Transfer flies to a new vial with food every 3-5 days, to prevent overcrowding and to easily identify virgin progeny (if desired).

***Note:*** Flies in the assay may be control strains (*Canton-S, w*^*1118*^, etc.) or progeny from Gal4/UAS crosses, including crosses for optogenetic experiments (see protocol below).

13. Ideal sample collection conditions:
  a. 20-25 flies per group, aged 3-7 days, is often sufficient sample size for most applications of the flyPAD/optoPAD assay.
    i. Run a power analysis if desired to confirm adequate sample size.

**CRITICAL:** Do not use CO_2_ within 24 h of beginning the flyPAD experiment, as this can influence fly behavior. Instead, use a fly aspirator to transfer flies into the appropriate arenas.

### Preparing experimental food for flyPAD and/or optoPAD assays

**Timing: 1 week prior to experiment; 15 min**

The flyPAD/optoPAD system allows presenting any two types of substrates to the fly, provided that the substrates can be prepared in a stable solid or semi-solid form that maintains consistent contact with the electrode surface throughout the assay. While agar-based matrices are commonly used to ensure structural stability and prevent spreading or drying, the system is not limited to agar-only formulations. Each substrate type used in our laboratory, including its specific ingredients, preparation instructions, and storage conditions, is described individually below.

In addition to standard sucrose-based food, the system is compatible with a range of modified laboratory diets, including yeast-based, high-sugar, high-protein, and nutrient-restricted formulations, as long as they are embedded in a stable matrix suitable for behavioral measurement. Sucrose-based food is provided as the primary example. Any deviations from this procedure (steps omitted or added) for other food types are indicated in Table 2.

14. Preparation of sucrose food
  a. To a microwave-safe beaker, add 10 mL of hot distilled water (up to 95°C), 0.70 g sucrose (200 mM) and 0.07 g Agar (0.7%). For consistency, place the mixture on the mug warmer on 55-75°C, stirring every 15 second until all components are completely dissolved.
  b. Pour sucrose food into a 5 mL Eppendorf tube, filled approximately half-way (approximately 2.5 mL).
  c. This will create 4 tubes of food, which yields ∼8 experiments.
  d. Ensure food solidifies within 1 hour at RT.
15. Food can be stored at room temperature (21°C) for approximately 2 weeks or reheated twice.

**Table 2:** Experimental flyPAD/OptoPad food recipes

Food type	Ingredients	Assay
Yeast and Sucrose	10 mL distilled water 7% (0.70 g) yeast 200 mM sucrose (0.70 g) 0.76% (0.076 g) Agar	Total consumption
Yeast (protein-rich)	10 mL distilled water 7% (0.70 g) yeast 0.7% (0.07 g) agar	Choice (open-loop) Learning (closed-loop)
Sucrose (sugar)	10 mL distilled water 200 mM sucrose (0.70 g) 0.7% (0.07 g) agar	Choice (open-loop) Learning (closed-loop)
Quinine (bitter)	2.5 mL 2 mM quinine stock (0.016 g in 20 mL water) in 7.5 mL distilled water to make 0.5 mM quinine solution 0.7% (0.07 g) agar	Choice (open-loop)

## Key resources table

**Table:** 

REAGENT or RESOURCE	SOURCE	IDENTIFIER
**Chemicals, peptides, and recombinant proteins**		

Agar, pure, powder	Thermo Scientific	400402500
D-Sucrose	Fisher BioReagents	BP220-1
Yeast flakes	Genesee Scientific	Cat#62-106
Quinine Hydrochloride Dihydrate	Sigma-Aldrich	22630
All trans-retinal	Sigma-Aldrich	R2500-1G
All trans-retinal	Spectrum Chemical Mfg. Co.	R3041-1GM
Propionic acid	Fisher Scientific	A258-500
Tegosept	Genesee Scientific	Cat#20-258

**Software and algorithms**		

MATLAB R2024 software	The Mathworks, Inc.	https://www.mathworks.com
Bonsai	Manufacturer	www.bonsai-rx.org
BioRender	BioRender	https://biorender.com
GraphPad Prism 10 software	GraphPad software	https://www.graphpad.com/features

**Other**		

FlyPAD 4-fly arena module	Itskov *et. al,*^13^	www.flypad.rocks
LED optogenetic add-on module	Itskov *et. al,*^13^	www.flypad.rocks
LED controller optogenetic add-on module	Itskov *et. al,*^13^	www.flypad.rocks
FlyPAD feeding multiplexor	Itskov *et. al,*^13^	www.flypad.rocks
Rapid food dispensing system, Droso-Filler™	FlyStuff	www.flystuff.com
Flystuff *Drosophila* vials	Genesee Scientific	32-113
Droso-Plugs	Genesee Scientific	902064
Beaker 250 mL	Fisher Scientific	50-199-7455
5 mL Eppendorf tubes	Genesee Scientific	24285
Pipette tips	Genesee Scientific	24121R
Eppendorf Research plus 1-channel	Fisher Scientific	TI13690034
Mug warmer	Amazon	amazon.com
Microwave	Any standard supplier	amazon.com
Cheese cloth	Genesee Scientific	53-100
Plastic wrap	Fisher Scientific	15-610
Kim wipes/lab tissue	Genesee Scientific	88115
Scissors	Amazon	amazon.com
Mouse pads	Amazon	amazon.com
Aluminum foil	Fisher Scientific	01-213-102

**Experimental models: *Drosophila* strains**		

*D. melanogaster*: fox-l3/TM6B	Christie *et al.*,^1^	N/A
*D. melanogaster*: UAS-Kir2.1	Baines *et al.,* 2001	BDSC: 6595
*D. melanogaster*: UAS-CsChrimson	Klapoetke *et al.*, 2014	BDSC: 55134
*D. melanogaster*: empty-split-GAL4	N/A	BDSC: 79603

## Materials and equipment

**Timing: Acquire reagents, consumables, and equipment above 1–2 months prior to planned experiments**

Table 1 features more detailed information regarding the flyPAD and optoPAD equipment purchased from the manufacturer. For a complete list of food recipes used in our laboratory, see Table 2.

## Step-by-step method details

This section lists major steps to run several different applications using the flyPAD and optoPAD systems, including preparing data files prior to the assay (steps 1–3), running choice and total consumption behavior using the flyPAD (steps 4–13) and optoPAD (steps 14–17), a closed-loop experiment driven by the flies own behavioral input (steps 18–21), and subsequent data analysis and processing (steps 22–25). Finally, we provide analytical pipelines for analyzing data collected from the total consumption (steps 26–27) choice (steps 28–30) and closed-loop (step 31).

### Preparing data files prior to assay

**Timing: 5 min prior to assay**

Each flyPAD arena module consists of 8 recording channels, and experimental layouts are therefore structured in multiples of 8 channels per multiplexed unit. This modular organization is important when combining multiple flyPAD units via the feeding multiplexor, as total channel number and file indexing must remain consistent across all connected modules. Accordingly, experimental designs and data acquisition files should always be configured such that the total number of arenas corresponds to an integer multiple of 8 (e.g., 8, 16, 24, etc.), ensuring correct channel mapping and downstream data alignment during analysis.

**Figure 4:**
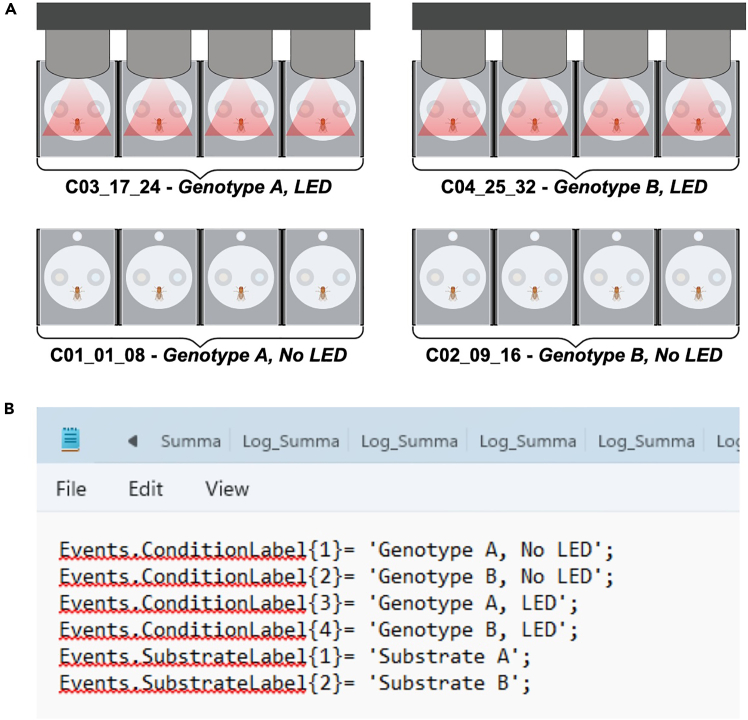
Example of Fly/optoPAD choice assay setup and corresponding log file (A) Schematic of a flyPad experiment with four experimental conditions looking at two different genotypes (Genotype A or B) and two illumination states (LED on or off). Each flyPAD channel contains one of two food substrates (e.g., yeast on the right, sucrose on the left) with one fly loaded per chamber. Continuous LED illumination (e.g., optogenetic stimulation) is applied to the top row of chambers, while the bottom row remains unilluminated. Each genotype is tested under both LED conditions (example shown: n=4 per condition). Condition labels (C0#) correspond to entries in the Bonsai and log files. The numeric ranges (e.g., 01_08) indicate the channel numbers assigned to each condition. Channels are numbered from left to right, starting with 01 at the bottom left. (B) Example log file corresponding to the experimental setup in (A) to be used as a reference for downstream MATLAB analysis. Events.ConditionLabel {#} specifies the genotype and LED condition for each group. This may also include additional factors such as: fly sex, mating status, starvation condition, etc. The number of condition labels in the log file must match those defined in the Bonsai file. Events.SubstrateLabel {1} and {2} indicate the substrates loaded on the left and right channels of each chamber, respectively. If only one substrate is tested, only SubstrateLabel {1} is required. Created in BioRender. Endres, M. (2026) https://BioRender.com/wjqk994.

***Note:*** As shown in the “MatrixWriter” node, the sensor number starts from 0 and not 1. However, for downstream analysis purposes, the file should be named according to naming conventions above.

1. Establishing a directory for saved data.
  a. Open the “Bonsai” software on the acquisition PC.
  b. Choose the relevant protocol you wish to run.
  c. Double click on “matrix-writer”.
    ***Note:*** A panel should open up to the right on the screen.
  d. Where it says “path”, select the ellipsis.
  e. Select the directory where you want your data file to be saved.
    ***Note:*** It is recommended to save each experiment in its own folder.
2. Naming the files (see Figure 4)
  a. Files will be named according to experimental conditions (number of groups and number of flies per group).
    i. For example, C01_01_08_C02_09_16. This would suggest that you have two groups (C01 and C02).
    ii. Each one of those groups will utilize 4 chambers (01_08) refers to individual flyPAD channels, with 01 and 02 referring to channels within the first chamber, 03 and 04 referring to channels within the second chamber, etc.

3. Preparing a text/log file for metadata
  a. In the experimental folder, make a.txt file
  b. Label each group by the following conventions:
    i. Events.ConditionLabel{1} = ‘Group 1 information’
    ii. Events.ConditionLabel{2} = ‘Group 2 information’
  c. Label each substrate by the following convention:
    i. Events.SubstrateLabel{1} = ‘food pipetted on the LEFT in each chamber’
    ii. Events.SubstrateLabel{2}= ‘food pipetted on the RIGHT in each chamber’

**CRITICAL: 1:** How you label the data file indicates where each group is located and what conditions they are exposed to for the analysis. However, files can be renamed after the experiment is run.

**CRITICAL: 2:** Be sure to remember and log which food of interest is pipetted where and keep this consistent *within* each experiment. You may alternate food sides *between* experiments.

### Behavior protocol: flyPAD assay

**Timing: 15-min preparation, plus 50-min experiment, with 15 min of cleanup/reset**

The flyPAD experiment allows the fly to freely feed between two choice points. This type of experiment is ideal for studying.

a. Total consumption: baseline feeding behavior on a standard food.
e. Choice: baseline choice feeding behavior between two different types of food.

**Figure 5:**
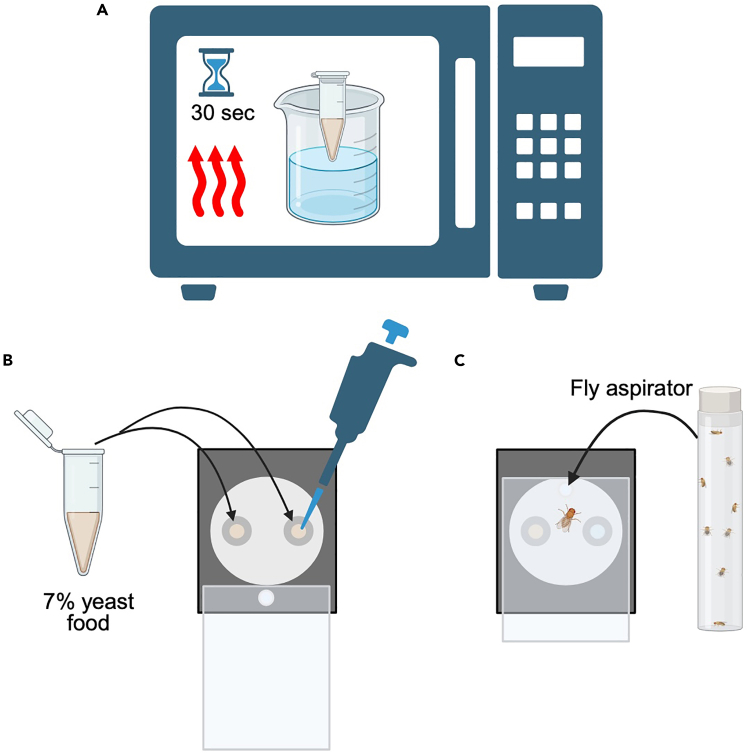
General procedure for open-loop flyPAD assay to measure total consumption (A) Food media is liquified by microwaving for ∼30s in 10s intervals. The media should not reach a boiling state. The media is then maintained in a hot water bath to prevent solidification prior to loading. (B) Approximately 3 μL of food media is pipetted into each flyPAD channel, taking on a dome-like shape. For total consumption measurements, identical media is loaded into both channels. (C) A single fly is loaded into each flyPAD chamber using a fly aspirator through a small opening in the transparent top panel. The chamber is then fully sealed, and the assay can begin. Created in BioRender. Endres, M. (2026) https://BioRender.com/j7nrrtf.

**Figure 6:**
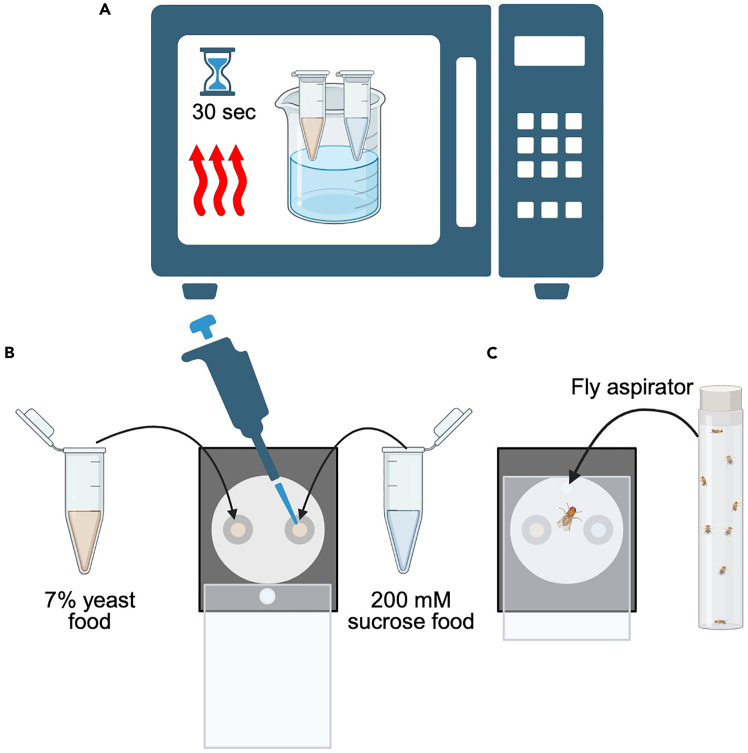
General procedure for open loop flyPAD assay to measure two-choice (A) Each food media is liquified by microwaving for ∼30s in 10s intervals. The media should not reach a boiling state. The media are then maintained in a hot water bath to prevent solidification prior to loading. (B) Approximately 2-3 μL of food media is pipetted into each flyPAD channel, taking on a dome-like shape. For choice experiments, one media type should be consistently loaded on the left channel, and the other media on the right channel. (C) A single fly is loaded into each flyPAD chamber using a fly aspirator through a small opening in the transparent top panel. The chamber is then fully sealed, and the assay can begin. Created in BioRender. Endres, M. (2026) https://BioRender.com/rsqzwyw.

For example, you may wish to determine the total amount of food a particular fly line consumes over the 50-minute testing period (Figure 5), or if the fly prefers protein vs sucrose (Figure 6).

4. Pre-Experiment PC preparation
  a. Turn on the PC that is associated with your flyPAD rig.
  b. Open up the “Bonsai” software.
  c. Select your protocol of choice, in this case choose an open-loop based protocol.
  d. Create a directory and.txt file as directed above.
5. Warming food for the assay
  a. Place 5 mL tubes of sucrose and protein on a microwave-safe tray, with the lids open.
  b. Fill a beaker (200 mL) with ∼150 mL of water.
  c. Microwave the tubes and the beaker for approximately 1 minute.
    i. While microwaving check every 15-20 seconds.
    ii. Sucrose and protein will be done when they are in liquid form and warm to the touch.
  d. Place tin foil over the water-filled beaker and push the tubes with your food through to the other side. Store tubes in warm water (see Figure 1).
    i. Place the beaker with tubes on the mug warmer at 55°C during experiments. You may also use a heat block if available.

***Note:*** This is to keep the food warm and liquified while you are working with it.

**CRITICAL:** You do not want the food to solidify too early in the paradigm, as it makes it difficult for the flies to consume. Ensure that the food is indeed warm, in a liquid state, and the water remains warm to the touch while working with it.

6. Loading food into flyPAD channels (Substrate A; Left Channel)
  a. Set a 10 μL pipette to 10 μL.
  b. Load desired food into the LEFT channel within each arena.

***Note:*** Each channel doesn’t get a precise amount of food. Your goal here is to provide a base layer of food. We recommend ∼3.33 μL into each (3 chambers per pipette full).

**CRITICAL:** While pipetting the food, you should not be getting any food on the brass section of the arena, as this will interfere with accuracy of results and require more troubleshooting (see below).

7. Loading food into flyPAD channels (Substrate B; Right Channel, see Figure 5).
  a. Repeat step 3 for your alternate (two-choice assay) or same (consumption assay) substrate for the right channel.

***Note:*** If running a total-consumption experiment, you should load the same substrate in BOTH channels (Figure 6).

**CRITICAL: 1**: The sucrose food is transparent, so avoiding the brass becomes more difficult. As such, you may put a 1:200 ratio of blue dye into the sucrose food if necessary. Our lab has determined no significant differences with fly feeding between sucrose or sucrose with dye containing foods (data unpublished but available upon request). Alternatively, you can detach each flyPAD module and pipette food under 4X magnification using a light microscope and return them to the behavioral testing location.

**CRITICAL: 2:** Please ensure you are changing your pipette tip after different foods to prevent cross-contamination.

8. Add an additional layer of food of each substrate to create a dome shape.
  a. Pipette 1.25 μL of substrate A and B over each (respectively).
  b. See Figure 7 for an illustrative example.

**Figure 7:**
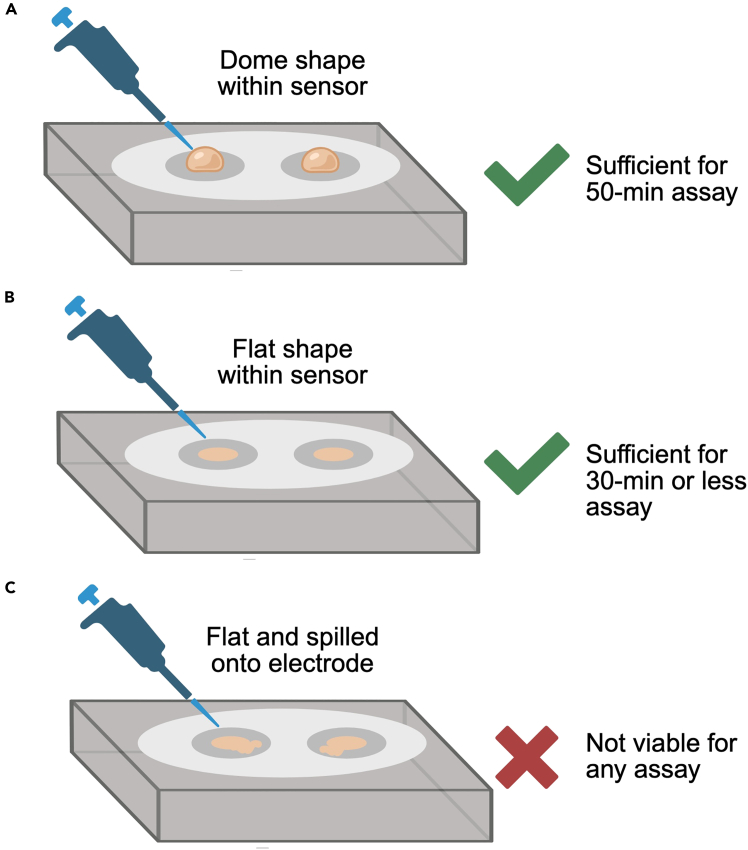
Proper loading of food media into assay chambers Using a micropipette, dispense ∼2-3 μL of liquified food media into the center of each channel. In longer assays spanning 50 min, the media should form a raised, dome-like meniscus (A), which enables effective interaction with the food by the fly. In shorter assays spanning 30 min or less, food can take on a flat shape within the channel, without spilling onto the sensor (B). If the media spreads into a flat layer that spills onto the electrode (C), the data will not be viable for any assay. If this occurs, wipe the sensor with a lab tissue and add ∼1 μL of additional media to the top until a domed shape is achieved. Created in BioRender. Endres, M. (2026) https://BioRender.com/1eqhdru.
9. Pre-Experimental Validation
  a. See step 4 of the setting up and procurement of the flyPAD Units and optoPAD LED modules section.
  b. Once you confirm all capacitance lines are moving and wavy, select “stop” on the bonsai software.
    i. If any are flat, wipe up excess food using a lint-free cloth and re-check.

**CRITICAL:** If an entire row is horizontal, this indicates a likely connection issue with that particular choice equipment and the rig. Please see troubleshooting section below.

10. Load flies into each arena using a fly aspirator. Ensure you load flies as per your experimental conditions.
  a. To load flies:
    i. Partially open each arena.
    ii. Tilt your fly aspirator such that the fly is pushed toward the back of the arena.
    iii. Immediately close chamber shut when flies are loaded.
11. Starting the experiment
  a. Hit “start” on the bonsai software to initiate the experiment.
  b. Set a lab timer for 50 min.
  c. Ensure the chambers are exposed to minimal to no lighting (darkness preferable)
  d. If you wish to run multiple experiments in the same day:
    i. Place the protein and sucrose food onto the mug warmer, set to 55°C.
    ii. Alternate sides of presented food between experiments as a control.
12. Ending the experiment
  a. After 50 min, press “stop” in Bonsai to end the experiment.
  b. Re-collect the flies for additional tests or discard them based on institutional parameters.
13. Clean-up and resetting for another experiment
  a. To clean, use a sterile 10 μL pipette tip and insert it into each choice hole to push the food out.
  b. Use lightly damp lint-free tissue paper with distilled water to wipe up the residual food. You should also do this on the underside portion of the arenas.
  c. Once complete, ensure the arenas are dry using lint-free tissue paper and clear of all food residue.

### Behavior protocol: optoPAD open-loop assay

**Timing: 15-min preparation, plus 50-min experiment, with 15 min of cleanup/reset**

This section provides detailed methodology on performing a total consumption or choice experiment, where an experimenter wishes to determine the flies’ baseline consumption, or preference after neuronal activation or inhibition of food consumption between two different food types. This type of experiment is ideal for studying.

a. How activation or inhibition of specific cells impacts feeding behavior in real-time.

For example, you may wish to determine whether your particular fly line has a preference for protein or sucrose when a subset of neurons or glia cells are activated or inhibited with the LED module. Optogenetic stimulation in our lab is delivered via a red optoPAD LED module (other colors may be used for different channelrhodopsins), which consists of externally mounted LEDs positioned above each desired fly arena. During setup, LED modules are placed directly over the corresponding arenas to ensure uniform illumination across the behavioral chamber. During setup, ensure the orientation of the optoPAD module is correct (the large ribbon cable should be extending out the back of the unit). Each LED channel is independently controlled through the optogenetic LED controller, allowing assignment of stimulation parameters on a per-arena basis.

**CRITICAL:** Ensure flies have been raised on retinal food (1:500) or placed in a collection retinal vial (1:250) for 3 days prior to beginning the experiment.

14. Repeat steps 1 (PC preparation) through 6 (pre-experimental validation) above from the “flyPAD assay” protocol. Use either the total consumption or two-choice formats as desired.
15. When loading flies for optoPAD:
  a. You should include a non-LED control, or a subset of flies not exposed to light. See Figure 4 for an example experimental schematic.
  b. Example of groups:
    i. Non-LED - variable A level 1
    ii. Non-LED - variable A level 2
    iii. LED- variable A level 1
    iv. LED- variable A level 2
16. Situating the optogenetic modules for stimulation
  a. Place the provided optogenetic module(s) over each desired LED exposed arena.
  b. Turn the LED electronic module “ON”. You can set the voltage based on what percent LED stimulation you would like.
    i. EX. 3V: 50%
  c. Cover the non-LED groups using mousepads or similar to avoid any light saturation.

**CRITICAL SAFETY WARNING:** Do not look directly into the LED lights as this may cause visual impairment.

17. Repeat steps 8 (starting the experiment) through 10 (cleaning up) from the above flyPAD total consumption/two-choice protocol.

### Behavior protocol: optoPAD closed-loop assay

**Timing: 15-min preparation, plus 50-min experiment, with 15 min of cleanup/reset**

**Figure 8:**
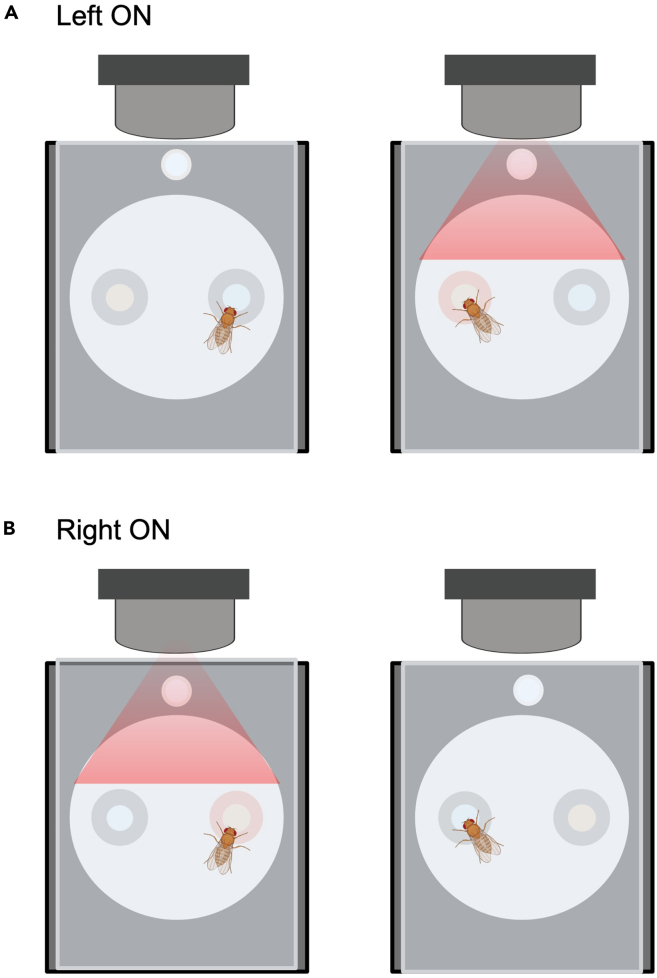
Closed-loop experiment pairing optogenetic neural manipulation with a specific food source In closed-loop experiments, the LED is paired with the electrode associated with one designated food type. (A) When the LED is paired with the left electrode, interactions with the right food source do not trigger illumination. However, when the fly interacts with the left food source, the LED is activated to induce optogenetic neural manipulation. (B) Conversely, when the LED is paired with the right electrode, interactions with the right food source activate the LED to induce optogenetic neural manipulation, while interactions with the left food source do not trigger illumination. Created in BioRender. Endres, M. (2026) https://BioRender.com/p5hngrt.

In a closed-loop experiment, the fly’s own behavior is used as an input to modulate system output. For example, you may perform an experiment whereby every time a fly takes a sip of a particular substrate, there is consequential LED delivery, and real-time circuit manipulation. When synchronization between light and food consumption is achieved on the left side: the LED light turns on when the fly drinks from the left, and remains off on the right, and vice versa. See Figure 8 for an example of the optoPAD closed-loop experiment format.

As opposed to open-loop, the closed-loop behavior is best to test.

a. If flies can learn to avoid or prefer a particular food option.
b. If neural activity alters decision-making at the moment of choice.
18. Pre-Experiment PC preparation for “Left ON” experiment
  a. Open the Bonsai software.
  b. Select the OptoPAD program, “Optopad_B2_4_NewVersion_Blinking _v2”.
  c. When saving a directory in the “MatrixWriter” node:
    i. Create a new folder named “closedloop_date”.
    ii. In this folder, create two subfolder files, one “LEFT ON” and the second “RIGHT ON.”
    iii. Use naming conventions for conditions and flies per arena as described above.
19. Setting the LED to only trigger in the left channel (“Left ON”)
  a. Open the “Device 1” node in Bonsai.
  b. Right click and select “Properties”.
  c. Under file name, select “Protocol”.
  d. Click on the ellipsis that appear on screen, and select the “Left ON” protocol provided from the manufacturer.
20. Repeat “Behavior protocol: flyPAD assay” steps 2 (warming food) through 10 (cleaning up and resetting above).

**CRITICAL: 1:** Wells should likely contain the same tastants, but this depends on experimental hypotheses and the research question.

21. Run “Right ON” experiments by following steps 1-3 above, except switching left and right channel instructions.

***Note:*** It is recommended to run both “Left ON” and “Right ON” experiments for each condition you plan to test, and averaging results to account for any intrinsic side bias.

***Note:*** You may also run the experiment as both LED’s ON if your experimental design would benefit from this configuration.

### Data processing

**Timing: Any time post-experiment; 10 min**

Data will be processed using data analysis software provided by the manufacturer. Below, we will describe a workflow using MATLAB, but the same data handling and analytical concepts can be applied to other software. We will start from processing the raw data and conclude with recommended statistical applications.

22. Preparing the directory for data analytical software.
  a. Open the desired directory on the PC containing your saved data.
  b. There will be two data files and your.txt file.
    i. The larger file (∼50-60 MB) is your data file from the 50-minute experiment, and the one you need for analysis.
    ii. Ensure it is named appropriately as described above.
    iii. The smaller file (1-5MB) is your pre-experimental validation file. This data file needs to be deleted and/or removed from this directory.
23. Data analytics
  a. Open your desired analytical software (MATLAB).
  b. Navigate to the manufacturer-provided flyPAD analysis directory and execute the analysis script.
  c. When asked, “do you want to load existing data file”, select “NO” if you want to analyze a new file.
  d. When asked, “do you want to take all conditions without reordering them”, select “YES”.
  e. Select the appropriate hardware configuration.
    i. Choose 32 flies for the sample data or two-choice arenas (2016-2017).
    ii. Choose 64 flies if you are using a one-choice arena (2016-2017).
    iii. Choose 48 flies if you are using a two-choice arena flyPAD (2018-present)∗.
    iv. Choose 96 if you are using a one-choice arena (2018-present)
  f. When asked, “do you want to load the log file”, select “YES”.
  g. Navigate to the directory where your.txt file is saved (see section, preparing a text/log file for metadata) and open it.
  h. When prompted to edit the content of the log file, click “OK” after verifying accuracy.
  i. A panel labeled “Conditions” will automatically open. At this condition prompt:
    i. Set the duration (360000 duration in samples, 100 S/Sec corresponds to 60 min).
    ii. Set the bins for the cumulative feeding measurements (1000 corresponds to 10 seconds).
    iii. All other parameters are recommended by the manufacturer to remain unchanged.
  j. When asked if you want to plot the results, click “YES” and wait for the conclusion of the analysis. This will take less than 5 min.
24. Navigating to save data and preparing the data file for analysis
  a. Navigate to the experimental folder(s) analyzed above.
  b. Open the newly created “mrep” folder, which contains output files created by the MATLAB workflow.
  c. Open “DatainExcelFormat”.
  d. Data will be organized in several tabs (Number of sips, Sip Duration, Activity Bouts). containing output metrics described below.
  e. In the excel file, rows represent individual flies, and columns represent specific experimental conditions as labeled in the.txt file (I.E., Group A, LED, substrate 1; Group A, LED, substrate 2).
  f. Prior to statistical analysis:
    i. Verify that column labels correctly match the.txt file for your experiment.
    ii. Verify that no channels have a sip count of 0, often indicating error while calibrating the channel or non-eaters. Remove these flies from analysis.
    iii. Remove any known mislabeled groups or hardware failure.
25. Output metrics from the assay are described in Table 3, with analytical applications included in Table 4.

**Table 3:** Data output and interpretation

Output	Description	Interpretation/Use
Spill Quantity	Fraction of time of the experimental recording that the signal was saturated due to contact with the top electrode	Reflects food contacting electrodes due to improper loading or flies spilling the food during feeding.
Removed Non Eaters	Number (or proportion) of flies excluded because they did not meet a minimum feeding threshold	Flies that never fed or fed below a defined cutoff (2 or less activity bouts on both electrodes during the whole duration of the experiment).
Sip Durations	Duration (ms) of a single feeding contact	Proxy for ingestion strength or palatability. Together with Inter Sip Interval define the frequency of feeding (feeding rhythm)
Inter Sip Intervals (ISI)	Time between consecutive sips within a feeding episode	Short ISI = sustained feeding; long ISI = disengagement.
Number of Sips	Total count of feeding events per fly	Proxy for total intake
Activity Bout Number	Number of episodes of active exploration of food sources	Feeding initiation frequency
Activity Bout Duration	Median duration of active exploration of food sources	Sustained engagement with food
Feeding Bursts Number	Number of feeding bursts per fly. A feeding burst is a sub-behavior within an activity bout, characterized as rapid sequences of sips with very short ISIs	Micro-pattern of ingestion
Feeding Bursts Duration	Duration of each feeding burst	How intensely flies feed within bouts
Activity Bout IBI	Time between the end of one bout and the start of the next	Reflects satiety, motivation, or learning
Feeding Burst IBI	Pause between feeding bursts (within a bout).	Short feeding burst IBI’s may be interpreted as increased motivation for a particular food source.

**Table 4:** Calculated metrics from raw output, their description, and interpretation

Metric	Calculation	Description	Interpretation
Preference Index	(SipA-SipB)/(SipA + SipB)	Relative feeding choice between two substrates	1.0 = A preference 0.0 = No preference -1.0 = B preference
Vigor	Number of Sips/Sip Duration	Measure of feeding intensity (tempo)	Higher values indicate stronger engagement with the substrate, higher urgency
Average sips per activity bout	Number of Sips/Activity Bouts	Measure of feeding continuity	Higher values indicate longer, sustained engagement with the substrate

### Analytical pipeline: Total consumption data

**Timing: Anytime post-experiment; 1–2 h**

Below are specific instructions for analyzing a flyPAD or optoPAD experiment where a fly was presented the same food substrate in both channels, and the experimenter wishes to examine total consumption differences in the substrate between genotypes or other experiment-specific variables. Here we provide an example for an experiment whereby an experimenter wishes to compare two genotypes (A and B) on total food consumption (sugar and yeast-based media). Running a MATLAB script to merge channel 1 and 2 for each fly is recommended, but you may also look at each channel’s feeding behavior to examine if there are any observed side biases. Table 5 demonstrates what raw data will look like in the MATLAB output excel file.

26. Analyzing data from a total consumption experiment involving 2 or more genotypes.
  a. Compare feeding events (sips) between two genotypes using an unpaired (independent-samples t-test).
  b. For experiments containing more than two genotypes, analyze data using a one-way ANOVA followed by appropriate post-hoc multiple-comparison tests with alpha correction.
27. Analyzing data from a total consumption experiment involving two or more genotypes and optoPAD (non-LED vs LED)
  a. For experiments containing two or more genotypes (A vs B) as well as a properly controlled optoPAD experiment (non-led vs led conditions), it is recommended to run a two-way ANOVA to model the interaction between the led status and genotype.

**Table 5:** Total consumption raw data

Genotype A: Number of sips	Genotype B: Number of sips
389	263
276	200
271	198
189	178

### Analytical pipeline: Choice data (creating a preference index)

**Timing: Anytime post-experiment; 1–2 h**

Below are specific instructions for analyzing a flyPAD or optoPAD experiment where a fly was presented with a choice between two substrates. In addition to compare the number of sips, a preference index can be calculated for comparison between genotypes or conditions.

A preference index, as opposed to looking at raw number of sips, has several advantages. Due to several extraneous variables not limited to but including fly genetics, time of day, vial conditions, and/or housing parameters, there may be baseline differences in feeding behavior. A preference index normalizes the data to allow more interpretable comparisons between experiments.

$$A\;preference\;index\;will\;take\;the\;form\;of:\frac{\left(NumberSipsA-NumberSipsB\right)}{NumberSipsA+NumberSipsB}$$

Such that:

1.0 = Full preference for Substrate A.

0.0 = No preference.

-1.0 = Full preference for Substrate B.

28. Calculating the Preference Index
  a. Open the “Number of Sips” panel within the excel document.
  b. For each group, apply the preference index formula above between your first and second substrate (Table 6).
    i. In the formula, substrate A is typically the substrate of interest.

**Table 6:** Two-choice assay raw data

Group 1, substrate A	Group 2, substrate A	Group 1, substrate B	Group 2, substrate B
456	345	283	383
673	309	118	258
173	286	51	489
430	76	386	226
29. Example for calculating a preference index (Table 7):
  a. Subtract each substrate A value from the substrate B value.
  b. Divide this by the addition of the total number of sips for both substrate A and B.

**Table 7:** Calculating a preference index for substrate A

Group 1, substrate A	Group 1, substrate B	Formula	Preference Index for A
456	283	(456-283)/(456 +283)	0.23
673	118	…	0.70
173	51	…	0.55
430	386	…	0.04
30. Statistical applications
  a. Analysis pipeline 1: Comparing the preference index for substrate A from group 1 and group 2 to examine group level differences in preference.
    i. Independent samples t-test (one group, two-levels).
      1. Ex. Male vs female flies on protein preference.
    ii. Two-way ANOVA (two groups, two-levels)
      1. Ex. Sex (male vs female) and starvation condition (fed vs starved) on protein preference.
  b. Analysis pipeline 2: Comparing the preference value vs 0 to examine the absolute preference of each group.
    i. One-sample t-test against 0.
      1. Ex. Despite group 1 (M = 0.10) having a significantly increased protein preference compared to group 2 (M = 0.0), both groups have no absolute preference (not significantly different from 0.0).
      ***Note:*** Use appropriate parametric or non-parametric statistical tests after assessing data normality and considering the structure of the experimental design.

### Analytical pipeline: optoPAD closed loop

**Timing: Anytime post-experiment; 1–2 h**

Below are specific instructions for analyzing data in an optoPAD closed-loop assay. In this experiment, the fly was presented with 1% agar solution in both the left and right channel. The configuration of the experiment is left “ON”, or right “ON” such that when the fly sips from the agar in an “ON” channel, the LED will activate neurons of interest. In contrast, the opposing channel is labeled “OFF”, which will not promote activation of the LED. This assay can determine whether activating neurons of interest while feeding from a designated channel will lead to a learned preference for that channel.

Table 8 consists of a subset of raw data of a closed-loop assay involving two genotypes.

**Table 8:** optoPAD closed-loop raw data

Genotype A “on”	Genotype A “OFF”	Genotype B “on”	Genotype B “OFF”
43	37	1354	181
24	3	523	105
89	138	824	351
25	113	1828	451
32	41	122	135

#### Preference index

Similar to the preference index in the two-choice assay, taking the raw sip counts above, we can create a learned preference index using the following formula:

$$Preference\;Index:\frac{\left(NumberSips\text{ON}-NumberSipsOFF\right)}{NumberSipsON+NumberSipsOFF}$$

Such that:

1.0 = Full preference for “ON”channel.

0 = No preference for either the “ON” or “OFF” channel.

-1.0 = Full preference for the “OFF” channel.

31. For statistical applications, you can apply any of the formulae outlined in the two-choice example above as deemed appropriate for your experimental design.

## Expected outcomes

Successful implementation of this protocol will generate high-resolution measurements of feeding behavior in individual *Drosophila*. The flyPAD and optoPAD systems can be used to quantify total food consumption, dietary preference, feeding microstructure, and behavioral responses to closed-loop optogenetic stimulation. Because feeding behavior is highly sensitive to physiological state and environmental conditions, experimental manipulations such as age, sex, mating status, nutritional history, temperature, and genetic background can produce measurable changes in feeding behavior. Table 9 summarizes representative outcomes reported in the literature and observed in our laboratory using the flyPAD/optoPAD workflows described here.

**Table 9:** State-dependent outcomes on feeding behavior

Variable	Expected outcome on feeding behavior	Reference
Age (young vs old)∗	Total feeding generally declines with age. Sucrose diet increases lifespan.^18^^,^^19^	Galenza et al., 2016 Wong et al., 2009
Sex (male vs female)∗	Females (mated) generally consume more total food, and more protein, than males.^20^^,^^21^	Camus et al., 2018 Vargas et al., 2010
Female mating status (unmated or mated)∗	Mated females generally have a higher preference for protein, and eat more, compared to virgins.^20^^,^^22^	Camus et al., 2018 Ribeiro & Dickson 2010
Male mating status	Mixed results. Virgin males generally have an increased protein intake, which declines after ejaculation, and then is restored.^20^^,^^23^	Liu et al., 2024 Camus et al., 2018
Nutritional history/diet∗	Starvation or dietary restriction, and macronutrient specific diets affects total feeding, choice behavior, fecundity, and fitness.^23^^,^^24^^,^^25^	Liu et al., 2024 Itskov & Rubeiro, 2013 Lee et al., 2008
Temperature	At lower temperatures, *Drosophila* prefers plant foods as opposed to yeast.^26^	Brankatschk et al. 2018
Genetic background∗	Common control lines (*Canton-S, W118, Oregon-R)* show baseline differences in feeding behavior.^19^^,^^20^^,^^25^	Camus et al. 2018 Wong et al. 2009 Lee et al. 2008

**Figure 9:**
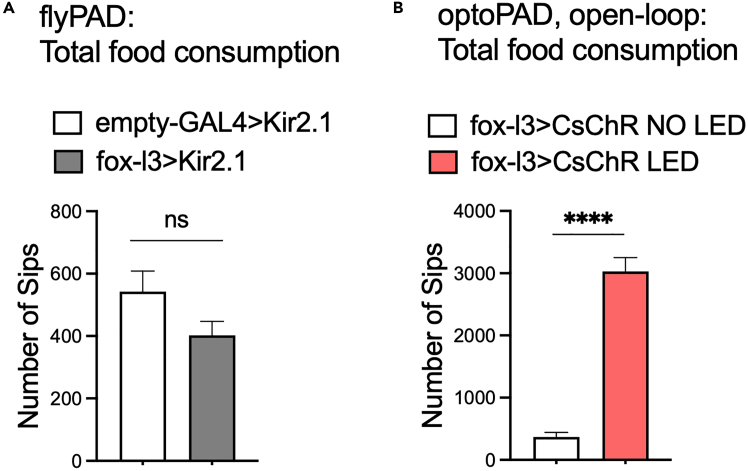
Example data for flyPAD total consumption In this example, we examined whether manipulating Fox neural activities affect total food consumption. (A) Silencing Fox neurons with Kir2.1 (fox-l3>Kir2.1) did not change fly’s total food consumption compared to the genetic control (empty-GAL4>Kir2.1). (B) Optogenetically activating Fox neurons (fox-l3>CsChR) significantly increased food consumption compared to the no LED controls. Data were analyzed using an independent samples *t* test, ns *p* > 0.05, ∗∗∗∗ *p* < 0.0001, error bars represent SEM.

**Figure 10:**
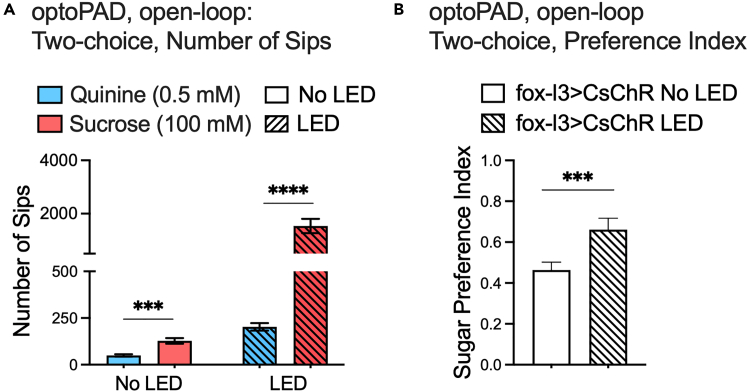
Example data for optoPAD open-loop two-choice with one genotype This experiment sought to examine whether activating Fox neurons (fox-l3>CsChR) affects fly’s preference between sucrose (100 mM) and quinine (0.5 mM). (A) fox-l3>CsChR flies showed innate preference for sucrose over quinine without optogenetic activation. Activating Fox neurons significantly increased fly’s consumption for both sucrose and quinine, shown as increased number of sips. (B) Preference index for sucrose shows that Fox activation further increased fly’s preference for sucrose. Paired *t* test was performed between number of sips for sucrose and quinine. Mann-Whitney test was performed on the preference index between no LED and LED groups. ∗∗∗*p* < 0.001, ∗∗∗∗ *p* < 0.0001, error bars represent SEM.

**Figure 11:**
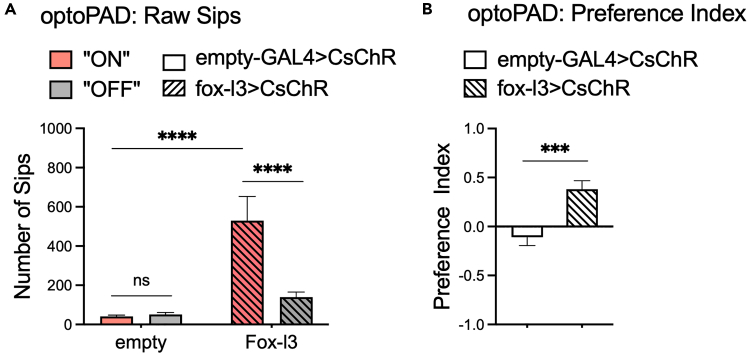
Example data for optoPAD closed-loop Closed-loop optoPAD experiments can be used to determine whether real-time LED activation, triggered by the fly’s own feeding behavior on the “ON” channel, promotes continued feeding from that channel. (A) Activation of Fox neurons (fox-l3>CsChR) significantly increased sips from the “ON” channel compared with the “OFF” channel. Flies with Fox activation also sipped more from the “ON” channel than empty-Gal4 controls, indicating that activating these neurons promotes feeding behavior. This effect was also observed when the data were normalized as preference index for the “ON” channel (B), with Fox activation producing a significantly higher preference for the “ON” channel compared with controls. Data from panel A were analyzed using a two-way ANOVA as described in our statistical methodology and followed up with Bonferroni adjusted post-hoc comparisons. Data from panel B were analyzed using an independent samples *t* test. N = 21-23 flies per group, error bars represent SEM. ∗∗∗ *p* <0.001, ∗∗∗∗ *p <*0.0001.

Our laboratory has recently used these approaches to identify a pair of neurons, termed Fox, that regulate total food consumption, dietary choice, and learned preference. Representative datasets generated using this protocol are shown in Figures 9, 10, and 11.

Figure 9 shows an example of how silencing or activating Fox affected total food consumption.^1^ Chronically silencing Fox with Kir2.1 (*fox-l3>Kir2.1*) did not affect baseline food consumption (Figure 9A), whereas optogenetically activating Fox with CsChrimson (*fox-l3>CsChR*) significantly increased food consumption (Figure 9B).

Figure 10 shows example data for fly’s choice between sucrose and quinine and how the preference is affected by activating Fox neurons.^1^ Flies showed preference for sucrose over quinine regardless of optogenetic activation of Fox (Figure 10A). To determine the effect of Fox activation, we calculated the preference index for sugar as described above and found that flies’ preference for sucrose was significantly increased by Fox activation (Figure 10B).

Figure 11 shows that activating Fox neurons induced a learned preference for the channel associated with Fox optogenetic activation, suggesting that Fox activation turns a neutral taste into a rewarding one.^1^ Control flies (*empty-GAL4>CsChR*) did not show a preference for either the “ON” or “OFF” channel, whereas flies with Fox activated (*fox-l3>CsChR*) showed significantly increased preference for the “ON” channel (Figure 11A). The difference between control and experimental groups is also revealed by the preference index for the “ON” channel (Figure 11B).

## Limitations

While the flyPAD/optoPAD offers several advantages, there are also some limitations worth noting. First, the assay allows for the choice between only two different food sources, which may not capture the full behavioral repertoire engaged in more complex feeding environment. Second, the arenas are designed primarily for testing individual flies and are not practical for assessing feeding behavior in group setting. Third, the assay is primarily optimized for short-term feeding measurements, typically within a 1 hour window. Therefore, experimenters should carefully consider the timing of the assay and potential effects of circadian rhythms, which are known to influence overall consumption patterns in *Drosophila*.^27^

## Troubleshooting

### Problem 1

Problem during steps 2–8 of “Behavior protocol: flyPAD assay”: Flies are not engaging with the assay or show very low feeding activity.

### Potential solutions

• If food has dried or solidified before or during the assay.
  ◦ Ensure assay food remains warm and liquid during loading and forms a dome-like shape in each well.
• If you suspect low feeding motivation.
  ◦ You may perform a 2-4 hour dry starvation or a 24–48 h wet starvation on a 1% agar based medium prior to the assay.
• If flies were exposed to CO₂ within 24 h of the experiment.
  ◦ Avoid CO₂ anesthesia within 24 h; transfer flies using a fly aspirator.
• If room temperature or humidity is outside the optimal range.
  ◦ Perform assays at 22–25 °C and ∼40–60% relative humidity.

### Problem 2

Problem during steps 2–8 of “Behavior protocol: flyPAD assay”: Food dries or runs out during the assay.

### Potential solutions

• For low ambient humidity.
  ◦ Ensure consistent (40–50%) room humidity. Use a local humidifier in room without central humidity control.
• For extended food preparation and loading time before experiment start.
  ◦ Practice sufficiently to ensure this time is minimized. We recommend 10 min from loading the food to starting the experiment at maximum.
• If certain food substrates dry faster than others.
  ◦ Load food in a consistent order and alternate sides between experiments.
• If the experimental time is too long.
  ◦ Ensure the assay is optimally 30–50 min in length, only.

### Problem 3

Problem during “Pre-experimental calibration and validation”: Flat/horizontal channels while starting assay.

### Potential solutions

• If the food is touching the brass electrodes.
  ◦ Remove excess food with a piece of lab tissue
  ◦ Repeat the pre-test until all lines are wavy/zig-zag

### Problem 4

Problem during “Initial hardware setup: flyPAD” and software configuration: Entire row of channels is inactive.

### Potential solutions

• For loose or improperly connected flyPAD arena.
  ◦ Ensure the connection to the COM port is secure.
  ◦ Verify the correct device is selected within Bonsai.

### Problem 5

Problem during “Initial hardware setup: optoPAD” and “Situating the optogenetic modules”: The LED lights are not functioning properly.

### Potential solutions

• If LED module is not properly seated or there are faulty connections.
  ◦ Reposition the module to ensure LEDs are securely attached.
• If the power supply turned off or is not properly set.
  ◦ Verify voltage output (3V or as desired) prior to starting the experiment.

### Problem 6

Problem during “Starting the experiment” and “Setting the LED to only trigger in the left channel”: Flies showing intrinsic bias for one probe/channel in a choice assay or closed-loop assay.

### Potential solutions

• If the chamber/arena is not level.
  ◦ Adjust the length of screws at corners to adjust the level.
  ◦ Ensure surface is level when placing flyPAD arenas.
• If light is not evenly distributed across the chamber.
  ◦ Ensure illumination is uniform and minimize light bleed in optogenetic experiments.
• If there is positional bias after optimization.
  ◦ Switch substances position in alternate trials and average the preference index of two back-to-back trials.

### Problem 7

Problem during “Preparing a text/log file for metadata” and “Data processing”: Issues with data analysis, error messages appear.

### Potential solutions

• If files are incorrectly formatted or labeled (log file or bonsai file).
  ◦ Verify file naming matches the arena layout and experimental conditions.
  ◦ Ensure the log file contains the correct groups and substrates.
  ◦ Ensure the “test” file used to troubleshoot the capacitance detectors is deleted from the directory.
  ◦ Ensure your length of experiment time entered matches how long the behavior was recorded.

## Resource availability

### Lead contact

Further information and requests for resources and reagents should be directed to and will be fulfilled by the lead contact, Lisha Shao (shaol@udel.edu).

### Technical contact

Technical questions on executing this protocol should be directed to and will be answered by the technical contact, Nicholas J. Collins (nicollin@udel.edu).

### Materials availability

All data provided in this manuscript were published in Christie et al.^1^ Raw data are available upon request form itskovpa@gmail.com.

### Data and code availability

For inquiries regarding the data analysis software and associated tools used with the flyPAD/optoPAD system, readers should consult the manufacturer documentation provided with the equipment. Additional requests can be made to Lisha Shao (shaol@udel.edu).

## Acknowledgments

We would like to thank Tarandeep Singh Dadyala, Sumaya Noor Sithy, and Laura White for helpful discussions and feedback on experimental design and protocol optimization. We would also like to thank the manufacturer, Pavel Itskov, for providing technical details when we were writing the manuscript. The graphical abstract was created in BioRender. Endres, M. (2026) https://BioRender.com/0v0yz0e. This work is supported by a Maximizing Investigators’ Research Award to L.S. (10.13039/100000057National Institute of General Medical Sciences, R35GM147504).

## Author contributions

All authors were involved with conceptualizing, planning, and reviewing all versions of the manuscript. N.J.C. wrote and edited the manuscript. M.N.E. made figures, resource tables, and edited the manuscript. I.T.S. made figures, resource tables, wrote and edited the manuscript. L.S. made figures, wrote, edited, and provided feedback on all versions of the manuscript. All authors have read and approved the final version of the manuscript.

## Declaration of interests

The authors have no conflict of interests to disclose.

## References

[1] Christie K.W., Dadyala T.S., Sinakevitch I.T., Chung P., Ito M., Shao L. (2026). A pair of interneurons that confer positive real-time valence to sweet sensation in Drosophila. Curr. Biol..

[2] Li W., Wang Z., Syed S., Lyu C., Lincoln S., O’Neil J., Nguyen A.D., Feng I., Young M.W. (2021). Chronic social isolation signals starvation and reduces sleep in Drosophila. Nature.

[3] Kelly K.P., Alassaf M., Sullivan C.E., Brent A.E., Goldberg Z.H., Poling M.E., Dubrulle J., Rajan A. (2022). Fat body phospholipid state dictates hunger-driven feeding behavior. eLife.

[4] Strilbytska O., Semaniuk U., Bubalo V., Storey K.B., Lushchak O. (2022). Dietary Choice Reshapes Metabolism in Drosophila by Affecting Consumption of Macronutrients. Biomolecules.

[5] Çoban B., Poppinga H., Rachad E.Y., Geurten B., Vasmer D., Rodriguez Jimenez F.J., Gadgil Y., Deimel S.H., Alyagor I., Schuldiner O. (2024). The caloric value of food intake structurally adjusts a neuronal mushroom body circuit mediating olfactory learning in *Drosophila*. Learn. Mem..

[6] Catalani E., Fanelli G., Silvestri F., Cherubini A., Del Quondam S., Bongiorni S., Taddei A.R., Ceci M., De Palma C., Perrotta C. (2021). Nutraceutical Strategy to Counteract Eye Neurodegeneration and Oxidative Stress in Drosophila melanogaster Fed with High-Sugar Diet. Antioxidants.

[7] Brüning J.C., Fenselau H. (2023). Integrative neurocircuits that control metabolism and food intake. Science.

[8] He L., Wu B., Shi J., Du J., Zhao Z. (2023). Regulation of feeding and energy homeostasis by clock-mediated Gart in Drosophila. Cell Rep..

[9] Pool A.-H., Kvello P., Mann K., Cheung S.K., Gordon M.D., Wang L., Scott K. (2014). Four GABAergic Interneurons Impose Feeding Restraint in Drosophila. Neuron.

[10] Rulifson E.J., Kim S.K., Nusse R. (2002). Ablation of Insulin-Producing Neurons in Flies: Growth and Diabetic Phenotypes. Science.

[11] Münch D., Goldschmidt D., Ribeiro C. (2022). The neuronal logic of how internal states control food choice. Nature.

[12] Pool A.-H., Scott K. (2014). Feeding regulation in Drosophila. Curr. Opin. Neurobiol..

[13] Itskov P.M., Moreira J.-M., Vinnik E., Lopes G., Safarik S., Dickinson M.H., Ribeiro C. (2014). Automated monitoring and quantitative analysis of feeding behaviour in Drosophila. Nat. Commun..

[14] Moreira J.-M., Itskov P.M., Goldschmidt D., Baltazar C., Steck K., Tastekin I., Walker S.J., Ribeiro C. (2019). optoPAD, a closed-loop optogenetics system to study the circuit basis of feeding behaviors. eLife.

[15] Deshpande S.A., Carvalho G.B., Amador A., Phillips A.M., Hoxha S., Lizotte K.J., Ja W.W. (2014). Quantifying Drosophila food intake: comparative analysis of current methodology. Nat. Methods.

[16] Ro J., Pak G., Malec P.A., Lyu Y., Allison D.B., Kennedy R.T., Pletcher S.D. (2016). Serotonin signaling mediates protein valuation and aging. eLife.

[17] Ja W.W., Carvalho G.B., Mak E.M., de la Rosa N.N., Fang A.Y., Liong J.C., Brummel T., Benzer S. (2007). Prandiology of Drosophila and the CAFE assay. Proc. Natl. Acad. Sci. USA.

[18] Galenza A., Hutchinson J., Campbell S.D., Hazes B., Foley E. (2016). Glucose modulates Drosophila longevity and immunity independent of the microbiota. Biol. Open.

[19] Wong R., Piper M.D.W., Wertheim B., Partridge L. (2009). Quantification of Food Intake in Drosophila. PLoS One.

[20] Camus M.F., Huang C.-C., Reuter M., Fowler K. (2018). Dietary choices are influenced by genotype, mating status, and sex in Drosophila melanogaster. Ecol. Evol..

[21] Vargas M.A., Luo N., Yamaguchi A., Kapahi P. (2010). A Role for S6 Kinase and Serotonin in Postmating Dietary Switch and Balance of Nutrients in D. melanogaster. Curr. Biol..

[22] Ribeiro C., Dickson B.J. (2010). Sex Peptide Receptor and Neuronal TOR/S6K Signaling Modulate Nutrient Balancing in Drosophila. Curr. Biol..

[23] Liu C., Tian N., Chang P., Zhang W. (2024). Mating reconciles fitness and fecundity by switching diet preference in flies. Nat. Commun..

[24] Itskov P.M., Ribeiro C. (2013). The Dilemmas of the Gourmet Fly: The Molecular and Neuronal Mechanisms of Feeding and Nutrient Decision Making in Drosophila. Front. Neurosci..

[25] Lee K.P., Simpson S.J., Clissold F.J., Brooks R., Ballard J.W.O., Taylor P.W., Soran N., Raubenheimer D. (2008). Lifespan and reproduction in Drosophila: New insights from nutritional geometry. Proc. Natl. Acad. Sci. USA.

[26] Brankatschk M., Gutmann T., Knittelfelder O., Palladini A., Prince E., Grzybek M., Brankatschk B., Shevchenko A., Coskun Ü., Eaton S. (2018). A Temperature-Dependent Switch in Feeding Preference Improves Drosophila Development and Survival in the Cold. Dev. Cell.

[27] Xu K., Zheng X., Sehgal A. (2008). Regulation of Feeding and Metabolism by Neuronal and Peripheral Clocks in Drosophila. Cell Metab..

